# Evaluating an integrated care pathway for frail elderly patients in Norway using multi-criteria decision analysis

**DOI:** 10.1186/s12913-021-06805-6

**Published:** 2021-08-28

**Authors:** M. Kamrul Islam, Sabine Ruths, Kristian Jansen, Runa Falck, Maureen Rutten-van Mölken, Jan Erik Askildsen

**Affiliations:** 1 Department of Economics, University of Bergen, Postboks 7802, 5020 Bergen, Norway; 2 Department of Social Sciences, NORCE Norwegian Research Centre, Bergen, Norway; 3 Research Unit for General Practice, NORCE Norwegian Research Centre, Bergen, Norway; 4 Department of Global Public Health and Primary Care, University of Bergen, Bergen, Norway; 5 Department of Nursing homes, Municipality of Bergen, Bergen, Norway; 6 Department of Comparative Politics, University of Bergen, Bergen, Norway; 7 School of Health Policy and Management, Erasmus University Rotterdam, Rotterdam, the Netherlands

**Keywords:** Frail elderly, Holistic assessment, Continuity of care, Integrated care, Mixed methods, Discrete choice experiment, Multi-criteria decision analysis, Multi-morbidity, Primary care, Quasi-experimental design

## Abstract

**Background:**

To provide value-based care for patients with multi-morbidity, innovative integrated care programmes and comprehensive evaluations of such programmes are required. In Norway, a new programme called “Holistic Continuity of Patient Care” (HCPC) addresses the issue of multi-morbidity by providing integrated care within learning networks for frail elderly patients who receive municipal home care services or a short-term stay in a nursing home. This study conducts a multi-criteria decision analysis (MCDA) to evaluate whether the HCPC programme performs better on a large set of outcomes corresponding to the ‘triple aim’ compared to usual care.

**Methods:**

Prospective longitudinal survey data were collected at baseline and follow-up after 6-months. The assessment of HCPC was implemented by a novel MCDA framework. The relative weights of importance of the outcomes used in the MCDA were obtained from a discrete choice experiment among five different groups of stakeholders. The performance score was estimated using a quasi-experimental design and linear mixed methods. Performance scores were standardized and multiplied by their weights of importance to obtain the overall MCDA value by stakeholder group.

**Results:**

At baseline in the HCPC and usual care groups, respectively, 120 and 89 patients responded, of whom 87 and 41 responded at follow-up. The average age at baseline was 80.0 years for HCPC and 83.6 for usual care. Matching reduced the standardized differences between the groups for patient background characteristics and outcome variables. The MCDA results indicated that HCPC was preferred to usual care irrespective of stakeholders. The better performance of HCPC was mostly driven by improvements in enjoyment of life, psychological well-being, and social relationships and participation. Results were consistent with sensitivity analyses using Monte Carlo simulation.

**Conclusion:**

Frail elderly with multi-morbidity represent complex health problems at large costs for society in terms of health- and social care. This study is a novel contribution to assessing and understanding HCPC programme performance respecting the multi-dimensionality of desired outcomes. Integrated care programmes like HCPC may improve well-being of patients, be cost-saving, and contribute to the pursuit of evidence based gradual reforms in the care of frail elderly.

**Supplementary Information:**

The online version contains supplementary material available at 10.1186/s12913-021-06805-6.

## Background

The global phenomenon of increasing life expectancy and proportion of elderly [1, 2] is also seen in Norway. The 2020 national population projections in Norway predict more elderly people in the population than previous projections. The share of persons aged 65 years or over will increase from 18 to 30% by 2070 [3]. Even though general health status of the elderly population in Norway has improved (for example, better self-reported health, being more physically active, and having less anxiety and depression), the demand for health care has not decreased during the last twenty years. There is little change in the number of people living with chronic disease or requiring assistance in activities of daily living [4] and resources spent in elderly care have increased [5]. The Organisation for Economic Co-operation and Development (OECD) projections predict that budgetary pressures in the coming decades are likely to come mainly from rising long-term care expenditure due to population ageing [6].

In Norway, hospitals are state-owned enterprises governed by appointed administrative boards. Municipalities are responsible for providing primary care and social services. Well-coordinated healthcare programmes can help the elderly with chronic conditions smoothly navigate the health and social care system. Within this endeavour, in 2012 the Norwegian Care Coordination Reform [7] was launched. An important intention of the reform was to develop coordinated clinical pathways across primary care and specialist care. The three primary objectives of the Coordination Reform are: (i) a more cohesive and coordinated approach to health and care services; (ii) a greater proportion of health and care services to be provided in the local communities; (iii) greater focus on preventative measures and improving public health.

Through the Norwegian Care Coordination Reform, the municipalities were given increased responsibility for community-based treatment, care and rehabilitation, to substitute specialized care by more affordable primary care [8]. A key challenge which the Care Coordination Reform intends to address, is that the common disease-specific approach in specialist care suffers from fragmentation and lack of person-centeredness when applied to people with multi-morbidity [9, 10]. As a result, generic clinical pathways for patients with multiple chronic diseases and frail elderly were developed, based on the involved municipalities’ experiences, as well as empirical research. Based on a previous model in the municipality of Trondheim by Røsstad and Grimsmo [11], in 2013 the Norwegian Ministry of Health and Care Services, the Norwegian Directorate of Health, the Norwegian Institute of Public Health and the Norwegian Association of Local and Regional Authorities developed generic clinical pathways for patients with multiple chronic diseases and frail elderly through the programme Learning network of “Holistic Continuity of Patient Care” (HCPC) [12]. Learning networks as a method is based on Breakthrough Series, developed in the USA in 1995 [13], in which teams from different health services gather in collaboratives for learning and improvement.^1^ The HCPC programme aims at improving patient pathways for the frail elderly by:

i) Moving attention from *“what is the matter with you”* to *“what is important to you”* so as to strengthen the service receiver’s role and contribute to equity and empowerment of the patient.
ii) More systematic collaboration between municipalities and hospitals.
iii) Follow-up of patient pathways using standardised measures.

The programme is targeted at frail elderly people living at home that have recent functional deterioration requiring additional municipal care services. The program involves early assessment, patient-centered follow-up, early involvement of the patient’s general practitioner (GP), and assigning a designated primary care contact.

There is a strong need for integrated care models [15, 16] for elderly people with multi-morbidity, as well as evaluations of such interventions and measures, in particular regarding the meso organisational and macro system-level care integration strategies [17–20]. Integrated care models for people with multi-morbidity are complex multi-faceted interventions that aim to improve a wide range of outcomes, often referred to as the Triple Aim (i.e. improving health/wellbeing, experience with care, costs [21]. In order to evaluate such models, a broad evaluation framework is required, in which a wide range of outcome measures are included. The current evidence of integrated care for frail elderly is inconsistent; the quality of studies is weak and they are inconclusive [22–24]. In particular, much of the previous studies evaluated the effectiveness and cost-effectiveness of these interventions for frail elderly by considering health outcomes such as functional limitations and health-related quality of life [24, 25]. These outcomes might not be appropriate for frail elderly people whose health is deteriorating [26, 27]. Hence, traditional cost-effectiveness studies seem unable to capture outcomes relevant to frail older people. Multi-Criteria Decision Analysis (MCDA) can provide a framework for evaluating integrated care on a broader set of outcomes [28, 29]. In MCDA the outcomes (i.e. criteria) can be reviewed separately but also integrated into one score by applying a relative weighting of importance to the outcomes. In addition, MCDA can consider multiple perspectives by using weights reflecting the preferences of different stakeholders [28, 30].

To our knowledge, no thorough scientific evaluation of patient outcomes related to introducing HCPC has so far been completed. Within the HCPC context, a qualitative exploration was conducted by Nilsen et al. [31]. They studied home care nurses’ experiences with implementation and systematic use of functional and wellbeing checklists developed for improving continuity of care and quality of care in the pathways provided to old and chronically ill patients in the communities. The checklists aimed to be person-centred and function-based as suggested by the findings from the Patient Trajectory for Home-dwelling elders (PaTH) project [32]. Using MCDA, this study aims to investigate the impact of introducing a specific model of integrated care for frail elderly patients, HCPC. We investigate specifically whether the HCPC programme contributes to improved health and well-being, experience of care and resource utilisation. The programme is evaluated as part of the European Union -financed SELFIE project (Sustainable integrated care models for multi-morbidity, delivery, financing and performance).^2^

## Methods

### Intervention

The three core differences between the HCPC programme (i.e. integrated care programme) and usual care (control group) are first the initial and follow-up (6 weeks) assessment of the patient’s level of functioning by validated tools; second the “everyday-rehabilitation” informed by the patient’s own goals for activities of daily living; and third the early involvement of the patient’s GP. As part of the programme a new professional role has been developed; a designated primary contact person (coordinator), notably a nurse or a social worker, responsible for individual patient follow up. The designated primary care contact works in the municipal care service. Focus is on functional ability rather than on disease and impairment. A patient’s GP is involved within 2 weeks after enrolment, i.e. through consultation at GP surgery or a home visit. The primary care teams comprise at least the patient’s coordinator and the GP. Other primary care professionals (e.g. physiotherapist, occupational therapist, social worker) are involved when appropriate. The HCPC and patient care involved are financed through the participating municipalities’ general budgets. There are no direct financial incentives towards municipalities.

### Population and data collection

The study population comprised frail elderly patients with multi-morbidity starting or extending their use of municipal home care services, or having a short-term stay in a nursing home, because of functional deterioration. While many of these patients were discharged from hospital recently, hospitalization was not mandatory for inclusion. The intervention group was recruited from municipalities participating in HCPC, and the usual care from municipalities not (yet) enrolled to this programme.

The HCPC group was derived from 12 municipalities in South- and Mid-Norway. Two of these municipalities were very small (< 2000 inhabitants), eight were small (5000 to 26,000 inhabitants) and two were mid-sized (44,000 to 46,000 inhabitants). The programme owner, The Norwegian Association of Local and Regional Authorities, introduced and recommended the study to the municipalities enrolled in HCPC through their contact network and during network meetings. Members of the research team attended several meetings, provided written and oral information to the municipalities and signed collaboration contracts. Primary contact persons in the enrolled municipalities recruited participants consecutively, i.e. they conducted home visits to eligible patients, provided study information, and collected informed consent. Within the eligible study population, there were no exclusion criteria. For patients with dementia or other conditions interfering with competence to give consent or answer survey questions, next of kin was invited instead. Three questionnaires at baseline and three at follow-up (for the same patients) were, and one questionnaire only at follow-up was completed by proxy. The enrolled patients’ primary contact person completed the “SELFIE-questionnaire for frail elderly” (we will refer to this questionnaire as the “SELFIE-Questionnaire” hereafter) based on a face-to-face patient interview in the patient’s home, at baseline (enrolment) and after 6 months. Data collection was conducted from September 2017 to June 2019.

The usual care group was derived from four municipalities in West-Norway not yet enrolled in the learning network programme of HCPC. Two of the municipalities were small (8000 to 16,000 inhabitants), one was mid-sized (30,000 inhabitants) and one was large (284,000 inhabitants). In the three small and mid-sized municipalities, research assistants (nurses/assistant nurses) conducted home visits to eligible patients, provided study information, and collected informed consent. In the large municipality, none of several attempts to recruite eligible home-dwelling people were successful. Instead, research assistants (employees/students at the University of Bergen, Norway) recruited eligible patients during short-term rehabilitation stays in nursing homes, before discharge to their own home. As with the HCPC group, there were no exclusion criteria in the usual care group. Next of kin was invited to fill out the questionnaire in cases where patients were unable to answer the questions by themselves. However, none of them filled out the questionnaire to a sufficient extent to be included in the study. For the enrolled patients, the research assistants completed the “SELFIE-Questionnaire” based on a face-to-face patient interview in the nursing home at baseline (enrolment) and in the patients’ home (after 6 months). Data collection was conducted from September 2018 to October 2019.

### Multi-criteria decision analysis (MCDA)

The SELFIE MCDA framework was developed based on established guidelines and follows the seven recommended steps: i) establish the decision-context, ii) identify and structure criteria, iii) determine the performance on criteria, iv) determine the weights of the criteria, v) create an overall value score, vi) perform sensitivity analyses, vii) interpret results [28, 29]. In *the first step* the aim is to establish what the likely decisions are that need to be made and thus how the MCDA will be used [28, 29]. Earlier qualitative study including document analyses and interviews with programme-initiators, managers, representatives of payer organisations and care providers of the HCPC programme has shown that the programme needs to provide evidence on the effectiveness of the intervention to help establish the long-term sustainability and potentially wider implementation of the programme throughout Norway [30, 33]. The aim of the MCDA was to inform these decisions quantitively by comparing the HCPC programme to usual care on the improvement in health and well-being, experience of care and resource utilisation. Moreover, it was important to identify the relevant stakeholders in this decision-making process. The stakeholders that were considered pertinent were five groups: Patients, Partners and other informal caregivers, Professionals, Payers, and Policy makers [33].

#### Outcomes/criteria (the second step)

The decision criteria that were used in the MCDA were the outcome variables that were measured with the “SELFIE-Questionnaire” specially developed for the integrated care programmes targeting elderly populations (see Appendix 1). Table 1 illustrates the validated items from well-established and widely used “instruments” for defining the outcomes included in the “SELFIE-Questionnaire”. All outcomes were related to one of the three domains of the Triple aim, i.e. health/well-being, experience of care and resource utilisation/costs (Berwick et al., 2008). A core set of outcomes included physical functioning, psychological well-being, enjoyment of life, social relationships and participation, resilience, person-centeredness, continuity of care and total health and social care costs.^3^ In addition, for the elderly patients, programme-specific outcomes included autonomy, informal care costs, long term institutional admission, and falls leading to hospital admissions. The distinction between a core set of outcomes and programme-specific outcomes was made within the SELFIE project, where the core set was measured in all evaluation studies as part of that project.

**Table 1:** Outcome measures included in the SELFIE-Questionnaire and their scale range

*Outcome*	*Validated instrument to measure outcome*
*Health/wellbeing*	
Physical functioning	Activities of daily living (Katz-15 for ADL); *0–15 (worst)* [53]
Psychological well-being	Mental Health Inventory (MHI-5); *0–100 (best)* [54]
Social relationships & participation	Impact on Participation & Autonomy (IPA), social life and relationships domain; *0–28 (worst)* [55]
Enjoyment of life	Investigating Choice Experiments for the Preferences of Older People (ICECAP-O); *1–4 (best)* [56]
Resilience	Brief Resilience Scale (BRF), *6–30 (best)* [57]
Autonomy^a^	Pearlin Mastery Scale (PMS) (*7–35) (best)* [58]
*Experience of care*	
Person-centeredness	Person-centered Coordinated Care Experience Questionnaire (P3CEQ), experience of person-centered care domain, *0–18 (best)* [59]
Continuity of care	Nijmegen Continuity Questionnaire (NCQ), team and cross boundary continuity domain + Client Perceptions of Coordination Questionnaire (CPCQ), item on waiting for appointment /treatment, *1–5 (best)* [60, 61]
Burden of medication^a^	Living with Medicines Questionnaire (LMQ), range 0–10 (worst) [62]
*Resource utilization and costs*	
Total health and care costs	Based on iMTA Medical Consumption Questionnaire (iMTA-MCQ) [35]
Informal care costs^a^	SELFIE-Questionnaire
Long term institutional admission^a^	SELFIE-Questionnaire
Falls leading to hospital admissions^a^	SELFIE-Questionnaire (ICD-10 code W00-W19)

^a^Included in the programme-type specific set of outcomes

To estimate and compare total health and care cost between HCPC and usual care, the cost components were identified and quantified from a societal perspective. The health and social care costs included the costs arising from the consequences of treatment (i.e. costs incurred with primary care services, e.g. GP and nurse, drug, and hospital costs) and formal social care costs. Costs related to informal care were estimated separately. Data on health, social care and informal care use were collected with an adapted version of the iMTA Medical Consumption Questionnaire and included within the “SELFIE- Questionnaire” [34].^4^ The questionnaire includes questions about contacts with GPs, primary care nurses, GP assistants, physiotherapists, dieticians, psychologists, dentists, social workers, welfare workers, and medical specialists, as well as hospital inpatient- and outpatient admissions, home care services, residential care and nursing homes, and informal care services during the past 3 months.

To quantify the total health and social care costs in monetary terms (Norwegian kroner - NOK) and to get the unit price of the relevant components, we used several different published documents and articles. In particular, national tariffs for GP services come from the Normal tariff for General Practitioners and Out-Of-Hours Emergency medical services 2018–19 document [36]. We converted all costs into 2019 prices using Consumer Price Index (changes in the price level of a weighted average market basket of consumer goods and services purchased by households) provided by Statatistics Norway (see [37]). The consultation fee for a GP was calculated as the weighted average of the fee for a non-specialist GP (NOK 165 (16.7 EUR^5^)) and an approved GP specialist (NOK 257 (26 EUR)). In estimating actual health care provider cost, out-of-pocket costs (co-payments) were assumed to be 30% of actual provider cost [39]. Per-diem in-patient hospital cost information was gathered from a recent published study from Norway [40]. Using their estimation, inflated by the Consumer Price Index, we estimated the average costs of a general ward bed day in Norwegian hospitals at NOK 8400 (853 Euros in 2019 price).

To estimate the medication costs we have collected the Defined Daily Dose, i.e. the assumed average maintenance dose per day for a drug used for its main indication in adults, by using the Defined Daily Dose Index 2020 [41]. The medication’s price was obtained using the joint directory for drugs [42]. The unit cost for social care and home care inpatient and outpatient components were gathered from reports published by the Norwegian Health Directorate [43, 44]_._ To estimate the informal care, we used relevant unit cost for home care services. The details on unit costs for social care and home care services by different municipalities are provided in Table A1 in Appendix 2.

#### Statistical analysis of performance scores (the third step)

The performance scores on the decision criteria of the MCDA were defined as the scores on the outcome measures described above. When estimating causal effects of using observational data, it is crucial to reduce systematic differences in the empirical distribution of the baseline (pre-intervention) confounders [45]. We performed a quasi-experimental approach called inverse probability of treatment weighting (IPTW) using the propensity score, to minimise the impact of any potential selection bias between HCPC and usual care at baseline. Logit regression was used to estimate the propensity score in which treatment status was regressed on selected observed baseline socio-demographic characteristics: age, gender, living condition, smoking status, multi-morbidity status (number of health problems≥2), and the baseline values of two core-set outcome variables, namely physical health, and social relationships and participation. The IPTW assigns a weight to each UC patient based on the similarity of the patient to the HCPC patients. In the IPTW the weights of the HCPC patients were set to 1 and the weights of the usual care patients were calculated with the formula *Weight = propensity score/(1–propensity score)*.

The baseline differences between the HCPC and usual care patients were assessed before and after the IPTW. Overall matching results were assessed by examining and reporting three test statistics. First, the mean (median) absolute standardised bias (i.e. the mean/median of the ratios of the difference of the sample means in the HCPC and usual care groups over the square root of the average of the variances in both groups); second, Rubin’s B, defined as the standardized difference of the means of the linear index of the propensity score in HCPC and usual care group, and third, Rubin’s R, defined as the ratio of HCPC and usual care variances of the linear index of the propensity score [46]. Statistical significance of the standardised differences have also been presented for all covariates before and after matching.

We estimated the “average treatment effect on the treated” for all outcome measures on the IPTW data. To analyse the outcomes we used repeated measurement models with individual level random intercept effects (i.e we assumed that the coefficients were fixed but the intercept varied randomly). We used models assuming continuous outcomes for ease of interpretation. Formally, we estimated the following equation:

$$ {y}_{jt}={\beta}_0+{\beta}_1{I}_j+{\beta}_2{T}_t+{\beta}_3{I}_j\times {T}_t+\chi {X}_{jt}+{\psi}_j+{\varepsilon}_{jt} $$

where *y*_*ji*_ is the outcome for individual j at time t; *Ij* is a dummy variable categorising intervention group (variable equals to 1 if the observation is from the HCPC cohort) and *T*_*t*_ is a dummy variable for time (equals to 1 if the observation is from T_1_ period), respectively. The coefficient for ${\hat{\beta}}_3$ describes the treatment effect. *X*_*jt*_ includes individual j_th_ age at time period t. *ψ*_*j*_ is the random error term for the jth individual and *ε*_*jt*_ is the remaining error term for j_th_ individual observed in the t_th_ period.

To calculate performance scores we predicted the mean score of the HCPC group at 6 months based on the regressions results. In addition we calculated the mean score of the usual care group assuming they had the same baseline score as the HCPC group. In this way the calculated performance scores could be directly compared between the HCPC and usual care group. This was done separately for each outcome.

#### Weghting the criteria (the fourth step)

Relative weights for the different criteria (i.e., outcomes) among stakeholders were elicited in an online weight elicitation study among Norwegian patients, informal caregivers, professional care providers, payers and policy makers. In the weight elicitation study, a discrete choice experiment (DCE) was used to obtain weights for the core set of outcomes (for full details of how these preference weight were obtained see [30, 47]. Table 2 gives the relative weights of the outcomes included in the MCDA, and as shown, all five stakeholder groups put relatively high weights on enjoyment of life and the lowest weight on cost.

**Table 2:** Relative DCE weights of the core set of outcomes used in the Multi-Criteria Decision Analysis by type of stakeholder

Outcome	Stakeholder perspective				
	Patients (*N* = 158)	Partners (*N* = 156)	Professionals (*N* = 161)	Payers (*N* = 122)	Policy makers (*N* = 180)
*Health/wellbeing*					
Physical functioning	0.178	0.107	0.124	0.141	0.115
Psychological well-being	0.175	0.184	0.167	0.155	0.149
Social relations & participation	0.110	0.154	0.141	0.117	0.144
Enjoyment of life	0.249	0.273	0.263	0.263	0.243
Resilience	0.109	0.088	0.110	0.130	0.126
*Experience of care*					
Person-centeredness	0.047	0.064	0.055	0.042	0.058
Continuity of care	0.110	0.112	0.106	0.092	0.128
*Costs*					
Total Health and Social care costs	0.023	0.017	0.034	0.060	0.035

Numbers in parentheses (N) by stakeholders indicate the number of participants included in the online weight elicitation study for the MCDA

#### Overall value calculation (the fifth step)

In the MCDA, the mean predicted outcome scores at T1 were first standardised on a 0–1 scale. This was done using relative standardisation with the equation:

$$ {S}_{aj}=\frac{x_{aj}}{{\left({x}_{aj}^2+{x}_{bj}^2\right)}^{1/2}} $$

Where *x*_*aj*_is the raw performance score in terms of mean predicted values for outcome j (on the natural scale) for the HCPC group and; ${x}_{aj}^2 and\ {x}_{bj}^2$ are the square of mean predicted values for outcome j for the HCPC group and the usual care group respectively. For all outcomes, the standardised score was set so that a higher score indicates better performance. To achieve this, in the above-mentioned equation, x was replaced by 1/x for reversely coded outcome measures (i.e outcomes where a higher score on the natural scale indicates a worse performance). We implemented an additive MCDA model where the standardised outcomes were weighted and subsequently summed to obtain a single overall value score for the HCPC group and a single overall value score for the usual care group.

#### Sensitivity analysis (the sixth step)

Probabilistic sensitivity analysis using Monte Carlo simulation was performed to evaluate the joint uncertainity of preference weights and performance scores. Cholesky decomposition was conducted for 10,000 replications to obtain a single overall value score. We calculated confidence (uncertainty) intervals around the overall value scores for each stakeholder group. The difference between the overall value score of the HCPC and the usual care is statistically significant if the confidence intervals do not overlap.

In the deterministic sensitivity analyses, we additionally conducted a swing weighting method for eliciting preferences [30, 48]. Within this analysis a wider range of outcomes (along with the eight outcomes in the core set), namely the programme-specific outcomes for frail elderly care programmes, were also included.^6^

All statistical analyses were performed with STATA 16.

## Results

### Sample characteristics

The study comprised information from 120 and 89 patients at baseline from the HCPC and usual care group respectively, and 86 patients in the HCPC group and 41 patients from usual care group at follow-up after 6 months. After scrutinizing the data, we found that there were some missing observations (varied from minimum 1 to highest 10) for a few outcome variables. To be pragmatic and to consider all available patients responding at baseline, we imputed these missing data on the outcome variables by their mean values and by the HCPC and usual care groups. However, we omitted four patients who responded at follow-up only but not at baseline.

Table 3 provides descriptive statistics on baseline characteristics of patients for two groups, and for pre-and post-matching data. In particular, before matching, the average age of the elderly patients at baseline was around 80.0 years for the HCPC group, while the corresponding average age was higher for the usual care group, at 83.6 years. Overall, as illustrated in the matching statistics given in the lower panel of Table 3, matching led to an improvement in the comparability of the two groups. The statistics satisfied the required criteria with recommended ranges (Rubin [46] suggested that the value of B < 25; 0.5 < R < 2 for sufficient balance). In particular, mean (median) standardised bias was 27.9 (28.0) in the unmatched sample, and after implementing the IPTW using the propensity score approach in the matched sample it was reduced to 6.3 (3.5); Rubin’s B was 88.6 in unmatched sample and reduced to 22.8 for the matched sample, and Rubin’s R showed 0.79 in unmatched sample and changed to 0.91 in matched sample.

**Table 3:** Baseline characteristics before and after propensity score matching

Variable	**Before matching**					**After matching and weighting**		
	HCPC (*N* = 120)		Usual care (*N* = 89)		*p*-value for the St. diff	Usual care (*N =* 89)		*p-*value for the St. diff
	Mean/ Prop	Std. Dev.	Mean/ Prop	Std. Dev.		Mean/ Prop	Std. Dev.	
**Demographic**								
Age^a^, mean	79.87	9.924	83.59	7.831	**0.009**	80.90	8.852	0.732
Male^a^, %	0.367	0.484	0.551	0.500	**0.003**	0.364	0.483	0.858
**Living condition**^a^**, %**								
Alone	0.617	0.488	0.517	0.503	0.123	0.536	0.501	0.321
Living with others	0.350	0.479	0.438	0.499	0.151	0.438	0.498	0.292
Missing	0.008	0.091	0.011	0.106	–	0.005	0.074	–
**Education status, %**								
Low	0.367	0.484	0.393	0.491	0.508	0.392	0.495	0.865
Medium	0.375	0.486	0.326	0.471	0.305	0.393	0.481	0.803
High	0.242	0.430	0.258	0.440	0.708	0.208	0.423	0.557
Education Missing	0.017	0.129	0.022	0.149		0.007	0.082	0.325
**Smoking status**^a^**, %**								
Current smoker	0.142	0.350	0.090	0.288	0.249	0.186	0.391	0.595
Previous smoker	0.492	0.502	0.584	0.496	**0.033**	0.441	0.499	0.657
Never smoker	0.350	0.479	0.315	0.467	0.205	0.368	0.484	0.944
Smoke missing	0.017	0.129	0.011	0.106	0.389	0.005	0.074	0.318
**Health condition**^a^**, %**								
≥ 2 conditions	0.983	0.131	0.888	0.318	**0.003**	0.979	0.143	0.316
Health missing	0.033	0.180	0.000	0.000	–	0.000	0.000	–
**Core-set Outcomes**								
Physical functioning^a^	4.792	3.103	6.517	3.402	**0.005**	4.997	3.138	0.895
Psychological well-being	73.05	17.38	74.79	20.49	0.420	71.50	22.81	0.656
Social relations & participation^a^	8.077	4.301	6.517	4.549	**0.003**	7..856	5.066	0.629
Enjoyment of life	2.630	0.746	2.609	0.894	0.791	2.468	0.946	0.376
Resilience	20.11	4.560	20.61	5.383	0.628	20.04	5.612	0.897
Person-centeredness	11.342	4.092	11.276	4.544	0.631	10.64	4.853	0.538
Continuity of care	3.558	0.743	3.496	0.672	0.844	3.411	0.657	0.317
Total health and social care costs (NOK)	133,269	164,187	130,919	113,511	0.423	124,855	117,798	0.760
**Programme-specific outcomes**								
Autonomy	23.85	4.666	21.97	6.081	0.079	21.58	6.240	0.044
Burden of medication	2.713	2.602	1.977	2.436	0.045	2.774	3.056	0.913
Informal caregiver burden	22,723	100,989	6017	28,965	0.168	4955	23,606	0.077
**Statistics to assess matching**								
Mean (Median) Bias^a^	26.5 (23.1)					7.1 (5.6)		
Rubin’s B^a^	97.8					24.9		
Rubin’s R^a^	0.91					1.08		

^a^variables used in PSM; St.diff = Absolute Standardised Mean Difference, also called Absolute Standardised Bias

**Fig. 1:**
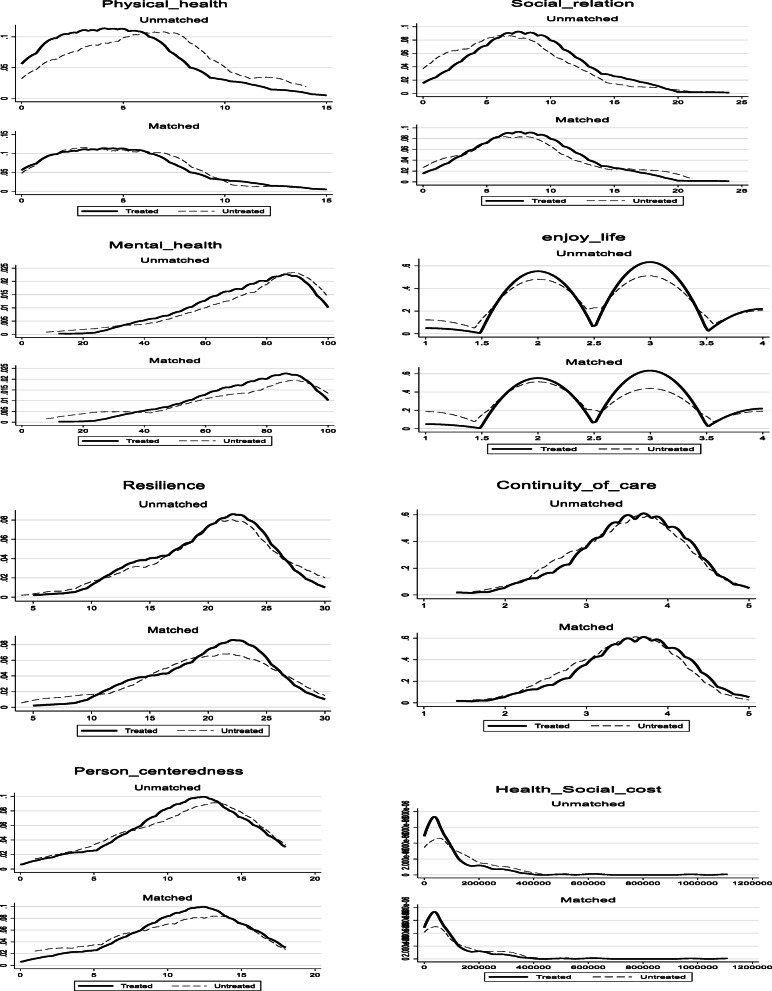
The distribution of the core outcome variables between HCPC (treated) and usual care groups (untreated) before and after matching

Figure 1 shows the distribution of the core outcome variables between the HCPC and usual care groups before and after matching. As is visualized, after matching the distributions are quite overlapping for all core-set outcomes provided in the figure.

#### Effects on outcomes

Table 4 illustrates the results of the statistical analyses of all core set outcomes included in the MCDA. The estimated “treatment effects on the treated”, ${\hat{\beta}}_3$ (coefficients of the interaction term between time and intervention), is provided in the last column of Table 4. The five health and wellbeing outcomes (the first 5 items/rows) consistently favour HCPC, and the experience outcomes favour UC, but there is no statistically significant effect (at the 5% level) of HCPC for any of these core-set outcome variables. A weak positive effect of the intervention is observed for the outcome variable *resilience*. A significant effect is observed for the programme-specific outcome *autonomy* (“*remaining in charge and making own decisions on how one lives his/her own life”).* The estimated coefficient indicates that HCPC negatively affects patients’ autonomy as measured by the Pearlin Mastery Scale (see Table 1), which is reduced by 2.9 points for the patients belonging to HCPC compared to usual care. A weak (at the 10% level) effect is observed for the *informal care cost* outcome in favour of HCPC.

**Table 4:** Health/Wellbeing and Experience Outcomes^d^: within and between group differences at 6 months

**Core set of outcomes included in the MCDA** (*N =* 336)	**Estimated change HCPC** **Mean**	**Estimated change usual care** **Mean**	**Diff. in change** **Mean** **(95% CI)**^**a**^
*Health/wellbeing*			
Physical functioning	−0.067	−0.041	−0.026 (−0.822; 0.769)
Psychological well-being	2.022	−2.803	4.825 (−7.380; 17.03)
Social relations & participation	0.608	1.163	−0.555 (− 2445; 1.335)
Enjoyment of life	0.163	−0.034	0.197 (−0.304; 0.697)
Resilience	0.555	−1.529	2.084^b^ (−0.080; 4.249)
*Experience of care*			
Person-centeredness	−0.374	−1.087	0.713 (−1.557; 2.984)
Continuity of care	0.025	0.078	−0.054 (− 0.297; 0.190)
*Cost*			
Health and social care cost (in Euro)	− 8170.3	− 5170.8	− 2999.4 (− 9607.6; 3608.8)
**Programme-specific outcomes included in the MCDA**^**d**^	**Estimated change integrated care** **Mean**	**Estimated change usual care** **Mean**	**Diff. in change Mean (95% CI)**^**a**^
Autonomy (*N =* 336)	−0.946	1.971	−2.917^c^ (−5.245; − 0.589)
Burden of medication (*N =* 328)	− 0.280	− 0.514	0.234 (− 0.758;1.226)
Informal care cost (in Euro) (*N* = 336)	− 1512.0	585.3	−2097.3^b^ (−41,713.0; 1002.2

^a^Based on robust SE; ^b^ and ^c^ indicate the level of significance at 10 and 5% respectively

Average exchange rate in 2019, 1 Euro = NOK 9.8527

^d^*A* negative sign means an improvement for Physical functioning and a postivive sign means a deterioration on social relationship and participation and burden of medication (see Table 1 for the the outcome’s scale range)

Table A2 (in Appendix 2) describes the health and social care costs differences for the last 3 months prior to interview date) by HCPC versus usual care at follow-up. The average total health and social care costs and informal care costs were lower for HCPC than usual care at follow-up. However, when categorising the total costs, we found that the costs of the care provided by the the GP, medical specialist and psychologist were *higher* for HCPC than usual care. Costs related to nurses, physiotherapists, hospital emergency care, hospital stay, inpatient and outpatient social care were substantially *lower* for HCPC than usual care.

As shown in Table 4, the treatment effect is also negative for health and social care costs (Euro − 2999), indicating that HCPC is less costly than usual care, as is the case also for costs of informal care. The estimate indicates a reduction in informal care costs of HCPC by Euro 2097 compared to usual care.

### MCDA overall scores

Table 5 reports the overall value score in the Multi-Criteria Decision Analysis (MCDA) for five Norwegian stakeholder groups (5Ps): *Patients, Partners, Professionals, Payers and Policy makers*. For All stakeholder groups, HCPC performed better than usual care, although the difference was small. Regarding the individual outcomes, the standardized performance score was higher for HCPC than usual care, except for continuity of care.

**Table 5:** Stakeholders’ value scores in the Multi-Criteria Decision Analysis

			Patients		Partners		Professionals		Payers		Policy makers	
Outcomes	Standardized performance score		Weighted score		Weighted score		Weighted score		Weighted score		Weighted score	
	HCPC	UC	HCPC	UC	HCPC	UC	HCPC	UC	HCPC	UC	HCPC	UC
Health/Well-being												
Physical functioning	0.708	0.706	0.126	0.126	0.076	0.075	0.088	0.088	0.100	0.100	0.082	0.081
Psychological well-being	0.730	0.683	0.128	0.119	0.134	0.126	0.122	0.114	0.113	0.106	0.109	0.102
Social relationships and participation	0.729	0.685	0.080	0.075	0.112	0.106	0.103	0.097	0.085	0.080	0.105	0.099
Enjoyment of life	0.732	0.681	0.182	0.169	0.200	0.186	0.192	0.179	0.192	0.179	0.178	0.166
Resilience	0.744	0.669	0.081	0.073	0.065	0.059	0.082	0.074	0.097	0.087	0.094	0.084
Experience of care												
Person-centeredness	0.730	0.683	0.034	0.032	0.047	0.044	0.040	0.038	0.031	0.029	0.043	0.040
Continuity of care	0.702	0.712	0.077	0.078	0.079	0.080	0.074	0.076	0.064	0.065	0.090	0.091
Costs												
Total costs	0.842	0.539	0.019	0.012	0.015	0.009	0.028	0.018	0.051	0.032	0.030	0.019
Overall value scores mean			0.728	0.685	0.728	0.685	0.730	0.682	0.733	0.678	0.730	0.682
Overall mean value scores including uncertainty [95% UI]			0.727 [0.727 0.728]	0.685 [0.684; 0.685]	0.727 [0.726; 0.727]	0.683 [0.683; 0.684]	0.729 [0.728; 0.729]	0.684 [0.684; 0.684]	0.731 [0.730; 0.731]	0.677 [0.677; 0.678]	0.747 [0.746; 0.747]	0.680 [0.680; 0.680]
Percentage HCPC > UC			94.8		94.9		94.7		95.1		98.7	

*HCPC* integrated care, *UC* usual care

### Sensitivity analysis

The robustness of the results was investigated by considering new sets of coefficients for treatment effect and importance weights using the Cholesky decomposition approach. The lower panel of Table 5 shows the uncertainty around the value score calculated with Monte Carlo simulation. For all five stakeholders, the MCDA results were very similar to our base analyses, indicating that the overall value scores were higher for integrated care than usual care. The proportion of the 10,000 simulations showing a preference for integrated care was between 94 and 99% for all stakeholders. The 95% confidence intervals *did not overlap.*

Table A4 in Appendix 3 also presents the value scores in the MCDA with the swing weights. The weights differed slightly, and the estimated score for the programme-specific outcome *autonomy* also here favoured usual care rather than HCPC. Overall, the MCDA results were highly consistent with our base analyses. The weighted scores were of similar magnitudes, and higher for integrated care than usual care for all five stakeholder groups.

## Discussion

### Summary of findings (the seventh step)

HCPC is an integrated care programme targeting frail elderly patients with multi-morbidity that have just started or extended their use of municipal home care services or have a short-term stay in a nursing home. The results from an MCDA, where a large set of outcomes corresponding to the ‘Triple aim’ (i.e. health/well-being, experiences with care, and costs) was taken into consideration, indicated that elderly patients in the municipalities participating in learning networks of HCPC fare better than elderly patients in the municipalities offering usual care. The result held for the perspectives of patients, partners, professionals, policy makers and payers. The differences in total MCDA scores between HCPC and usual care were constant over the five stakeholder groups. The main driver of this result seemed to be the difference in performance of the HCPC along the dimension *enjoyment of life*. Here HCPC improved patients’ experienceconsiderably more than usual care, although the effect was not statistically significant. There was little difference of opinion between the stakeholders with respect to enjoyment of life being the highest valued outcome measure. Furthermore, it appeared that the HCPC programme increased cost incurred for primary health care, but reduced comparatively costly components attributed to hospital stay and nursing home care, and cost associated with informal care. The municipalities cover the establishment costs for the HCPC programme, however, we did not have data on this intervention cost component (e.g. costs associated with intensified collaboration between sectors, costs of planning individual care pathways) at our disposal and it was not included in this analysis. Moreover, it is difficult to make any sensible assumptions on the costs related with collaboration between sectors and cost related with planning individual care pathways. We have been in contact with some of the municipalities to have them indicate costs associated without them being able to give us precise estimates. But they are not likely to be very high, since also individual pathways have to planned both in the HCPC and the usual care groups. Besides, when it comes to costs borne by the municipalities, who run the programmes, there is no strong indication of favourable or adverse financial incentives.

An interesting difference was observed when weighting outcomes with swing weights instead of DCE weights. Enjoyment of life was attached a lower weight, while autonomy was weighted quite highly. Furthermore, usual care patients fared better than HCPC patients when considering how they feel about mastering their own life. This may reflect that involving more health personnel in HCPC may provide better care but at the cost of more decision making left with professional care providers, which may negatively affect the patients’ feeling of autonomy.

### Strengths and limitations

The study is a novel contribution to investigating and understanding how an integrated health care programme performs when taking into consideration the multi-dimensionality of relevant outcomes. The SELFIE MCDA provides a structured and explicit evaluation framework for assesing an integrated programme with multiple health and wellbeing outcomes relevant to frail elderly patients. Even though we are aware of the methodological challenges discussed in the literature, particularly regarding which outcomes to include and how to assess uncertainty [49], the study provides some evidence that pure cost-effective analyses may miss important aspects of relevant outcome space. An international valuation study has shown that different stakeholder groups - patients, partners, professionals and policy makers - both within and between countries appreciated the enjoyment of life, social relationships and participation, and personal autonomy in addition to traditional health outcomes [47]. This study showed that on certain outcomes HCPC performed better than usual care and on others it did not. MCDA allowed us to use an explicit framework to aggregate the various outcomes and to determine an overall value score. Furthermore, we showed overall value scores from the perspectives of different stakeholders. The latter is important because aligning the preferences of different stakeholders is likely to contribute to the success of an integrated care approach. There is good reason to have confidence in the weights attached to the outcomes, obtained in a discrete choice experiment among a total of 776 stakeholders (~ 150 per stakeholder group) (Rutten-van Mölken et al., 2020).

Another strength is that the performance scores were estimated using a quasi-experimental framework (i.e. IPTW) by including a control group which provided better estimates of effectiveness than many previous studies in this field that are before-after studies. Nevertheless, the uncertainty around the overall value scores was formally incorporated using probabilistic sensitivity Monte-Carlo simulations on both weights and scores.

Methodologically, we have shown benefits from using an MCDA framework in evaluating care programmes aimed at patients that are in a situation with several, and often conflicting, demands. Hoverver, there are some methodological limitations with the MCDA framework discussed in the literature [49]. In particular, the creation of a composite measure of benefit without knowing the threshold value for the willingness to pay or the opportunity costs of one additional unit of that benefit (as researchers do know for QALYs); and whether cost should be consider as one of the criteria/outcomes in the MCDA. Questions could also be raised on the utility of the value scores from the different perspectives. Is there a perspective that should prevail? Or, should analysts average the perspectives? In our study we assumed that the performance scales and weight scales were linearly associated. Simplicity, transparency and implementational convenience favour the additive MCDA models. To avoid inappropriate double counting of value, the outcomes in an additive model should not overlap and the outcomes should be preferentially independent [49]. The potential violations of these key assumptions may not be ruled out in our additive MCDA framework. To study this may involve, for example, mapping between the discrete choice experiment levels and the performance scale. However, changing our assumption of linearity is unlikely to affect our MCDA results because the performance scores of the HCPC and usual care do not differ very much. Although the SELFIE MCDA framework has made efforts to overcome several challenges facing the approach (see [30]), future work could be attained on the actual use of the MCDA tool by decision makers to clarify how stakeholders want to engage with MCDA, and precisely when and how it contributes the most.

Further limitalions are connected with the patient recruitment and the impact evaluation. Patient recruitment in both HCPC and usual care municipalities was difficult for several reasons. It was particularly difficult to establish the usual care municipalities. The Norwegian Association of Local and Regional Authorities made efforts to recruit municipalities that were planning to join the HCPC programme, expecting these might have an interest in a comparative study. However, several municipalities declined, often due to a lack of resources to assist in collecting data, or general overload of work also related to planned merging of municipalities. Furthermore, although the HCPC group was easier to establish, patient recruitment here proved difficult since so many of the municipalities were small with few eligible patients. Another issue to consider was that frail elderly patients in general may have problems answering the lengthy questionnaire that was used for data collection on health and wellbeing, experience of care, and individual costs. For these patients, the follow-up interview was particulary problematic, since their health condition might worsen. Recall bias associated with health and social care, and informal care resource use could be a potential concern for the study using a retrospective self-reported questionnaire. However, such a bias would not be systematically different between the HCPC and usual care groups. Attrition rate as a result of losses to follow-up was relatively higher for the usual care group. At the 6-month follow-up, 41 patients from the usual care group (more than 46% sample at the baseline) responded, whereas 86 patients (around 72% of baseline sample) responded from the HCPC group. Ifloss to follow-up was systematically related to the patients’ underlying characteristics such as age, multimorbidity status etc., differences in the attrition rates for usual care and HCPC group could result in selection biases. High attrition rates are common in studies on elderly patients [50]. The HCPC group comprised exclusively home-dwelling frail elderly people, while the UC group was mainly recruited from short-term nursing home rehabilitation stays (93%) and mostly from one large municipality. The difficulty to recruit home-dwelling patients in this control municipality was attributable to the lack of dedicated primary care contact persons. This strategy may have introduced bias which we attempted to overcome through a successful implementation of the IPTW approach at baseline by controlling for the living condition attribute in estimating the propensity score. Moreover, different persons were involved in recruiting and interviewing patients in the different municipalities. Even though they were given instructions on patient selection and how to assist patients in answering, it is difficult to rule out reporting bias.

### Generalizability

The study population was derived from 16 municipalities of various sizes in different parts of the country, supporting transferability of the results to similar municipalities in other regions. The generic clinical pathways for patients with multi-morbidity in the HCPC programme ensured that the included patients have many different chronic conditions and thus comprised a representative population with multi-morbidity in need of municipal care services. Transferability of results to other countries depends on organisation and cooperation of health and social services, and how different outcomes would be weighted by different stakeholders.

### Comparison with existing literature

It is difficult to directly compare the MCDA results of the HCPC programme to other integrated care programmes. Many integrated primary care approaches targeting frail older persons have emerged over the years. As mentioned in the onset, however, the quality of previous studies was not good and evidence for their effectiveness and cost-effectiveness remained mixed [22–24, 51]. The integrated care programmes were highly multifaceted interventions and as such comparing them to each other introduced difficulties due to differences in interventions, outcomes and populations [52]. Nevertheless, in a recent comprehensive review, Baxter and colleagues [52] concluded that integrated care programmes/models might enhanced patient satisfaction, increased perceived quality of care, and enabled access to services, although the evidence for other outcomes including health and wellbeing outcomes and service costs remained uncertain.

### Implications for research and/or practice

The numbers of patients with multiple chronic diseases are increasing, which is a challenge to the public purse. To establish relevant and cost-efficient care it is vital to investigate the main needs and care demands of these patients. This project highlights the importance of providing care along different dimensions. The Norwegian HCPC programme for frail elderly patients is an integrated programme that has received much attention from the municipalities in Norway. The results of this study are important contributions to better understand the effects of integrated care programmes and may be useful for decision-makers at the municipal and central level for prioritisation of often-costly initiatives. Future research should focus on developing stronger links between outcomes and weights in the MCDA. Our results support furthering resources for a randomised controlled trial that may provide stronger conclusions on causality than possible in a quasi-experimental design.

## Conclusions

This study shows the importance of evaluating reforms and new initiatives for chronically ill and frail elderly patients, often with multi-morbidities, in a broad framework. Cost-effectiveness analyses may give a first-hand insight into the acceptability of spending resources on pure health and cost dimensions. However, the pure health state dimension does not capture all relevant features of what is relevant to these patients. The results clearly show the importance of a broad perspective such as the MCDA framework when considering care delivery in a transparent way. A national programme like HCPC, with moderate changes, may improve well-being of the patients, in the long term be cost saving, and overall be worthwhile pursuing in gradual care reforms for the frail elderly patients.

## Supplementary Information

**Additional file 1.**

**Additional file 2.**

**Additional file 3.**

## Data Availability

The stakeholder data used for weighing is anonymous (provided in Table 2), and can be available on request from the corresponding author (MKI). The data that support the findings provided in Tables 3, 4 and 5 are not publicly available due to them containing information that could compromise research participant privacy/consent.

## Abbreviations

MCDA: Multi Criterion Decision Analyses
OECD: The Organisation for Economic Co-operation and Development
HCPC: Holistic Continuity of Patient Care
DCE: Discrete Choice Experiment
PSM: Propensity Score Matching
IPTW: Inverse Probability Treatment Weighting

## Footnotes

1 Learning networks as a method has been internationally used and evaluated for a range of issues, evidence summary can be accessed [14]. In Norway, three national programmes have the short name “Learning network” (for children, for drug abuse/psychiatric care and for elderly).

2 Among other aims, the SELFIE project evaluates impact of 17 different integrated chronic care (ICC) programmes for patients with chronic conditions and multi-morbidities in eight European countries [30].

3 In the SELFIE project, several different integrated programmes were investigated. Some outcomes were common for all programmes, termed ‘core outcomes’, whereas other outcomes were specific for a smaller groups of treatment programs, like those involving frail elderly. The latter are termed ‘program specific outcomes’. The selection of the outcomes was based on a literature review, workshops with representatives from the five stakeholder groups, and focus groups with individuals with multi-morbidity [33].

4 The link to where people can order the questionnaires at [35].

5 Annual exchange rate for 1 euro = NOK 9.8527 in 2019 [38].

6 Table A3 in Appendix 3 shows the swing weights for five Norwegian stakeholders. We found some difference in the ranking of the weights over attributes and by stakeholders. For example, in swing weight, patients put relatively highest weight on physical functioning and then autonomy, while other stakeholders put relatively higher weights on enjoyment of life and autonomy.
