# Supplementary material for: Evaluating an integrated care pathway for frail elderly patients in Norway using multi-criteria decision analysis
Source: BMC Health Serv Res. 2021 Aug 28;21:884. doi: 10.1186/s12913-021-06805-6 (PMC8400755; doi:10.1186/s12913-021-06805-6)
Supplement: Supplementary file 1 — Additional file 1. [file 12913_2021_6805_MOESM1_ESM.pdf]

SELFIE
2020

SELFIE

Questionnaire

Instructions

This questionnaire asks questions about your health and well-being, your experience with care and your health care use.

We are interested in your opinion and your experience. There are no right or wrong answers. Choose the answer that best applies to your situation. Mark an X in the box of your choice.

Example

1. *Are you male or female?*
   - ☐ *male*
   - ☒ *female (you indicated that you are female)*

If you accidentally mark the wrong box, you can make the box black and mark the correct box.

2. *Are you male or female?*
   - ☒ *male (you have now indicated that you are male)*
   - ■ *female*

For our research, it is important that you answer all questions, even if you find them difficult. Your answers will remain strictly confidential, will not be shared with your care providers, and will not influence the care you receive.

Completion of the questionnaire will take approximately 25 minutes.

Many thanks in advance for completing the questionnaire.

Part I: Health and well-being

A2. Daily activities

The following items are about activities of daily living. Please mark for each question whether you need help to do these activities. For each question, please indicate if you need help with this activity. Please mark one box for each question.

		Yes	No
A2_1.	Do you need help with taking a bath or shower?	☐	☐
A2_2.	Do you need help getting dressed?	☐	☐
A2_3.	Do you need help toileting?	☐	☐
A2_4.	Do you need help sitting down and getting up from a chair?	☐	☐
A2_5.	Do you use incontinence products?	☐	☐
A2_6.	Do you need help with eating?	☐	☐
A2_7.	Do you need help using the telephone?	☐	☐
A2_8.	Do you need help shopping?	☐	☐
A2_9.	Do you need help preparing a meal?	☐	☐
A2_10.	Do you need help taking care of your house?	☐	☐
A2_11.	Do you need help travelling?	☐	☐
A2_12.	Do you need help taking your medications?	☐	☐
A2_13.	Do you need help handling your finances?	☐	☐
A2_14.	Do you need help brushing your hair or shaving?	☐	☐
A2_15.	Do you need help walking about?	☐	☐

B. How you feel

These questions are about how you feel and how you have been doing the past month. For each question, please mark the box that best applies to you. Please mark one box for each question.

How much of the time during the past month…		None of the time	A little of the time	Some of the time	A good bit of the time	Most of the time	All of the time
B1.	Were you a happy person?	☐	☐	☐	☐	☐	☐
B2.	Have you felt calm and peaceful?	☐	☐	☐	☐	☐	☐
B3.	Have you been a very nervous person?	☐	☐	☐	☐	☐	☐
B4.	Have you felt downhearted and blue?	☐	☐	☐	☐	☐	☐
B5.	Have you felt so down in the dumps that nothing could cheer you up?	☐	☐	☐	☐	☐	☐

C1. Enjoyment of life

Please indicate which statement fits your current situation best by marking ONE box.

☐ I can have all of the enjoyment and pleasure that I want
☐ I can have a lot of the enjoyment and pleasure that I want
☐ I can have a little of the enjoyment and pleasure that I want
☐ I cannot have any of the enjoyment and pleasure that I want

D. Social relationships

The next questions are about the quality and frequency of your social relationships. Please mark one box per question.

	Very poor	*Poor*	*Fair*	*Good*	*Very good*
D1. My chances of talking to people close to me on equal terms are	☐	☐	☐	☐	☐
D2. The quality of my relationships with people who are close to me are	☐	☐	☐	☐	☐
D3. The respect I receive from people who are close to me is	☐	☐	☐	☐	☐
D4. My relationships with acquaintances are	☐	☐	☐	☐	☐
D5. The respect I receive from acquaintances is	☐	☐	☐	☐	☐
D6. My chances of having an intimate relationship are	☐	☐	☐	☐	☐
D7. My chances of seeing people as often as I want are	☐	☐	☐	☐	☐

E. Resilience

Below are some statements. Please mark one box per statement.

	Strongly disagree	*Disagree*	*Neutral*	*Agree*	*Strongly agree*
E1. I tend to bounce back quickly after hard times	☐	☐	☐	☐	☐
E2. I have a hard time making it through stressful events	☐	☐	☐	☐	☐
E3. It does not take me long to recover from a stressful event	☐	☐	☐	☐	☐
E4. It is hard for me to snap back when something bad happens	☐	☐	☐	☐	☐
E5. I usually come through difficult times with little trouble	☐	☐	☐	☐	☐
E6. I tend to take a long time to get over set-backs in my life	☐	☐	☐	☐	☐

G. Autonomy (frail elderly)

Below are some statements. For each statement, please mark the box that best describes how much you agree. Please mark one box per statement.

	Strongly disagree	*Disagree*	*Neutral*	*Agree*	*Strongly agree*
G1. There is really no way I can solve some of the problems I have	☐	☐	☐	☐	☐
G2. Sometimes I feel that I am being pushed around in life	☐	☐	☐	☐	☐
G3. I have little control over the things that happen to me	☐	☐	☐	☐	☐
G4. I can do just about anything I really set my mind to do	☐	☐	☐	☐	☐
G5. I often feel helpless in dealing with the problems of life	☐	☐	☐	☐	☐
G6. What happens to me in the future mostly depends on me	☐	☐	☐	☐	☐
G7. There is little I can do to change many of the important things in my life	☐	☐	☐	☐	☐

Part II: Experience of care

J. Person-centeredness

The next questions are about your experience and understanding of the care you have received from your health and social care providers in the last month. 'Care' could be any treatment or support you received in relation to your health and well-being. Please provide a response based on your overall experience if you have received care from more than one service.

	Not at all	*To some extent*	*More often than not*	*Always*
J1. Did you discuss what was most important for YOU in managing your own health and well-being?	☐	☐	☐	☐
J2. Were you involved as much as you wanted to be in decisions about your care?	☐	☐	☐	☐
J3. Were you considered as a 'whole person' rather than just a disease/condition in relation to your care?	☐	☐	☐	☐
J4. Did your care team / providers involve your family/friends/carers as much as you wanted them to be in decisions about your care?	☐	☐	☐	☐

J5. Have you had enough support from your care team / providers to help YOU to manage your own health and well-being?

☐ I have had no support
☐ I have not had enough support
☐ I have had enough support
☐ I have had a lot of support

J6. To what extent did you receive useful information at the time you needed it to help you manage your health and well-being?

☐ I do not receive any information
☐ I rarely receive enough information
☐ I sometimes receive enough information
☐ I always receive enough information
☐ I receive too much information

K. Continuity of care

The following statements are about the collaboration between caregivers (for example between your general practitioner and the medical specialist in the hospital). For each statement, please mark the box that best describes how much you agree or whether the statement is not applicable (N/A). Please mark one box per statement.

	Strongly disagree	*Disagree*	*Neutral*	*Agree*	*Strongly Agree*	*N/A*
K1. My care providers transfer information very well to one-another	☐	☐	☐	☐	☐	☐
K2. My care providers work together very well	☐	☐	☐	☐	☐	☐
K3. My care providers are very well connected	☐	☐	☐	☐	☐	☐
K4. My care providers always know what one-another is doing	☐	☐	☐	☐	☐	☐
K5. I have to wait too long for an appointment or treatment	☐	☐	☐	☐	☐	☐

L. (L1) Burden of medication (frail elderly)

This question is about your general impression of your medicines. Put an 'X' on the line to indicate your opinion.

How much of a burden do you feel your medicines are to you? (Think for example of methods of administering, timely intake, side effects, understanding why you're taking them, concerns about combinations of medicines, and their costs)

0 1 2 3 4 5 6 7 8 9 10

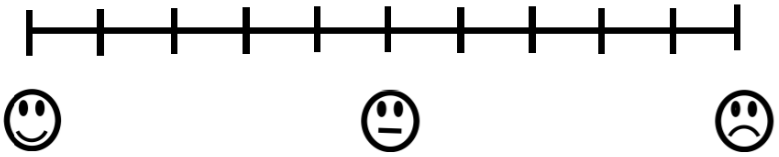

No burden at all

Extremely burdensome

Part III: Costs

N. Health and social care costs

The next questions are about your care utilisation in the last three months. Please add up all control visits, appointments, home visits, and consultations by telephone in the last three months. Telephone calls for making an appointment should not be counted. Please fill in how many consultations you have had approximately if you are unsure about the exact number of consultations.

	During the last 3 months, did you visit			
N1.	**........your general practitioner?**	☐ **No**<br>☐ **Yes, namely**	☐	**visit(s)**
N2.	**........a primary care nurse (e.g., practice nurse, nurse practitioner)**	☐ **No**<br>☐ **Yes, namely**	☐	**visit(s)**
N3.	**........a GP assistant?**	☐ **No**<br>☐ **Yes, namely**	☐	**visit(s)**
N4.	**........a physiotherapist?**	☐ **No**<br>☐ **Yes, namely**	☐	**visit(s)**
N5.	**........an occupational therapist?**	☐ **No**<br>☐ **Yes, namely**	☐	**visit(s)**
N6.	**........a speech therapist?**	☐ **No**<br>☐ **Yes, namely**	☐	**visit(s)**
N7.	**........a dietician?**	☐ **No**<br>☐ **Yes, namely**	☐	**visit(s)**
N8.	**........a podiatrist?**	☐ **No**<br>☐ **Yes, namely**	☐	**visit(s)**
N9.	**........a medical specialist (e.g., geriatrician, lung specialist, cardiologist, eye specialist, rheumatologist, neurologist, elderly care physician)** Add up all appointments.	☐ **No**<br>☐ **Yes, namely**	☐	**visit(s)**
N10.	**........a psychologist, psychiatrist or psychotherapist?** Add up all appointments.	☐ **No**<br>☐ **Yes, namely**	☐	**visit(s)**
N11.	**........a district nurse or community nurse?** Add up all appointments.	☐ **No**<br>☐ **Yes, namely**	☐	**visit(s)**
N12.	**........a social worker?**	☐ **No**<br>☐ **Yes, namely**	☐	**visit(s)**

N13.	**........a welfare worker?**	☐ **No**<br>☐ **Yes, namely** ☐ **visit(s)**
N14.	**........a homoeopathist or an acupuncturist?** Add up all appointments.	☐ **No**<br>☐ **Yes, namely** ☐ **visit(s)**<br>☐
N15.	**........dentist?**	☐ **No**<br>☐ **Yes, namely** ☐ **visit(s)**
N16.	**........optometrist?**	☐ **No**<br>☐ **Yes, namely** ☐ **visit(s)**
N17.	**........other, namely:** ☐	☐ **No**<br>☐ **Yes, namely** ☐ **visit(s)**
N18.	**........other, namely:** ☐	☐ **No**<br>☐ **Yes, namely** ☐ **visit(s)**
N19.	**........other, namely:** ☐	☐ **No**<br>☐ **Yes, namely** ☐ **visit(s)**

	During the last 3 months did you visit any of the following services below, and if so, how often? Please only fill in day-visits (outpatient), and not overnight stays (inpatient)	
N20.	**........residential care or nursing home**	☐ **No**<br>☐ **Yes, namely** ☐ **visit(s)**
N21.	**........rehabilitation center**	☐ **No**<br>☐ **Yes, namely** ☐ **visit(s)**
N22.	**........other, namely:** ☐	☐ **No**<br>☐ **Yes, namely** ☐ **visit(s)**

N23. **During the last 3 months, did you visit a hospital emergency room?**

☐ No → *Go to question H25*

☐ Yes, namely ☐ time(s)

N24. **Was the reason for your emergency visit a fall?**

☐ No

☐ Yes ☐ time(s)

N25. **Have you been admitted to a hospital in the past 3 months?**

☐ No → *Go to question H27*

☐ Yes, namely ☐ days in total *(add up all days in case of more admissions).*

N13.	........**a welfare worker?**	☐ **No**<br>☐ **Yes, namely** ☐ **visit(s)**
N14.	........**a homoeopathist or an acupuncturist?** Add up all appointments.	☐ **No**<br>☐ **Yes, namely** ☐ **visit(s)**<br>☐
N15.	........**dentist?**	☐ **No**<br>☐ **Yes, namely** ☐ **visit(s)**
N16.	........**optometrist?**	☐ **No**<br>☐ **Yes, namely** ☐ **visit(s)**
N17.	........**other, namely:** ☐	☐ **No**<br>☐ **Yes, namely** ☐ **visit(s)**
N18.	........**other, namely:** ☐	☐ **No**<br>☐ **Yes, namely** ☐ **visit(s)**
N19.	........**other, namely:** ☐	☐ **No**<br>☐ **Yes, namely** ☐ **visit(s)**

	During the last 3 months did you visit any of the following services below, and if so, how often? Please only fill in day-visits (outpatient), and not overnight stays (inpatient)	
N20.	........**residential care or nursing home**	☐ **No**<br>☐ **Yes, namely** ☐ **visit(s)**
N21.	........**rehabilitation center**	☐ **No**<br>☐ **Yes, namely** ☐ **visit(s)**
N22.	........**other, namely:** ☐	☐ **No**<br>☐ **Yes, namely** ☐ **visit(s)**

N23. **During the last 3 months, did you visit a hospital emergency room?**

☐ No → *Go to question H25*

☐ Yes, namely ☐ time(s)

N24. **Was the reason for your emergency visit a fall?**

☐ No

☐ Yes ☐ time(s)

N25. **Have you been admitted to a hospital in the past 3 months?**

☐ No → *Go to question H27*

☐ Yes, namely ☐ days in total *(add up all days in case of more admissions).*

N26. **Was the reason for your hospital admission a fall?**

☐ No

☐ Yes [ ] time(s)

N27. **Were you admitted elsewhere because of your health during the last 3 months?** *For example you stayed in a home for residential care or a nursing home, in a psychiatric hospital or rehabilitation center.*

☐ No → *Go to question H31*

☐ Yes

	During the last 3 months, I have been admitted to a…	
N28.	**……..residential care or nursing home**	☐ **No**<br>☐ **Yes, namely** [ ] **days in the past three months**
N29.	**……..rehabilitation center**	☐ **No**<br>☐ **Yes, namely** [ ] **days in the past three months**
N30.	**……..psychiatric hospital**	☐ **No**<br>☐ **Yes, namely** [ ] **days in the past three months**

N31. **Did you receive home care in the last 3 months?**

☐ No → *Go to question H32*

☐ Yes

	During the last 3 months (13 weeks), did you receive the following home care services…	
N32.	**……..housekeeping and home help** *(e.g., vacuum cleaning, doing the laundry, making up your bed, going for daily groceries)*	☐ **No**<br>☐ **Yes, namely** [ ] **hours a week**<br>[ ] **weeks during the last 3 months**
N33.	**……..personal care** *(e.g., help for bathing or help to get dressed)*	☐ **No**<br>☐ **Yes, namely** [ ] **hours a week**<br>[ ] **weeks during the last 3 months**
N34.	**……..nursing** *(e.g., put on a bandage, administer medicines, measure blood pressure)*	☐ **No**<br>☐ **Yes, namely** [ ] **hours a week**<br>[ ] **weeks during the last 3 months**

N35. **Did you take any medication during the last 3 months?**

☐ Yes

☐ No → *Go to question I1*

N36. **What medication did you take during the last 3 months?**

With medication we mean all drugs that were prescribed and medication that you have bought at the pharmacy or a drugstore. We have given 2 examples.

Note: *Filling in the questions below will be made much easier if you look at your medication boxes. You'll find the dose you have to take per time. And how often you have to do so per day.* ***Did you take more or less?*** *Please fill in the dose that you have actually taken.*

What is the medication name?	***How much did you take per time?***<br>*See the box or the package.*	***How many times did you take this per day?***<br>*See the box or the package.*	***How many days during the past 3 months did you take the medication?***
example 1<br>Metoprolol (blood pressure)	*example*<br>100mg	*example*<br>1 time	*example*<br>90 days
example 2<br>Furosemide (diuretic)	*example*<br>40 mg	*example*<br>1 time	*example*<br>26 days<br>(2 times a week; 13 weeks)

O. Informal caregiving (frail elderly and palliative care/oncology)

	Please indicate if in the past three months (13 weeks) you received the following help from family or other relatives due to your health condition. If yes, how often?	
O1.	**……..household activities?**<br>*For example, food preparation, cleaning, gardening, and taking care of and playing with your (grand)children*	☐ **No**<br>☐ **Yes, namely** ☐ **hours a week**<br>☐ **weeks in the past 3 months**
O2.	**……..personal care?**<br>*For example, dressing, washing, shaving, going to the toilet, eating, drinking and taking medication*	☐ **No**<br>☐ **Yes, namely** ☐ **hours a week**<br>☐ **weeks in the past 3 months**
O3.	**……..practical support?**<br>*For example, mobility outside the house including assistance with walking or wheelchair, visiting family or friends, seeing health care contacts (e.g., doctors' appointment), and taking care of financial matters*	☐ **No**<br>☐ **Yes, namely** ☐ **hours a week**<br>☐ **weeks in the past 3 months**

Part IV: Demographics

Q. Demographics

Q1. **What is the date today?**

D	D		M	M		Y	Y	Y	Y

Q2. **What is your date of birth?**

D	D			M	M		Y	Y	Y	Y

Q3. **What is your gender?**

- ☐ Male
- ☐ Female

Q4. **What is the highest degree or level of school that you have completed?**

- ☐ Early childhood education
- ☐ Primary education
- ☐ Lower secondary education
- ☐ Upper secondary education
- ☐ Post-secondary non-tertiary education
- ☐ Tertiary education
- ☐ Short-cycle tertiary education
- ☐ Bachelor's or equivalent level
- ☐ Master's or equivalent level
- ☐ Doctoral or equivalent level.

Q5. **What is your marital status:**

- ☐ Single (never married)
- ☐ Married / long-term relationship
- ☐ Widow / widower
- ☐ Divorced

Q6. **What is your living situation:**

- ☐ Independent, alone
- ☐ With others (partner, children, etc.)
- ☐ Care home / residential care centre since ……. /…….. /……. (DD/MM/YY)
- ☐ Nursing home since ……. /…….. /……. (DD/MM/YY)

Q7. **Which of the following statements about occupational status apply to you?** *(multiple answers allowed)*

- ☐ I have a paid job
- ☐ I do volunteer work
- ☐ I am retired or on pre-pension
- ☐ I am work disabled, for …………….. %
- ☐ I am looking for a job
- ☐ I have a paid job
- ☐ I am a housewife / househusband

☐ I am a student

Q8. **What is your smoking status?**

☐ Current smoker

☐ Former smoker

☐ Never smoker

R. Health conditions

The following is a list of common health problems. Please indicate for each condition if you have this or not. At the bottom there is space to list health problems not already presented.

Do you have this condition?	*No*	*Yes*
R1. Asthma	☐	☐
R2. Cancer during the past five years (not including small skin cancers)	☐	☐
R3. Chronic back pain or sciatica	☐	☐
R4. Chronic bronchitis, COPD or emphysema	☐	☐
R5. Chronic kidney disease	☐	☐
R6. Cognitive problems, memory loss, dementia, Alzheimer's disease	☐	☐
R7. Colon problem, irritable bowel or colitis	☐	☐
R8. Congestive heart failure	☐	☐
R9. Depression, anxiety or emotional difficulties	☐	☐
R10. Diabetes	☐	☐
R11. Hard of hearing	☐	☐
R12. Heart disease, angina (chest pain from heart problem), heart attack, bypass surgery or angioplasty	☐	☐
R13. High blood pressure	☐	☐
R14. High cholesterol	☐	☐
R15. Osteoarthritis (not rheumatoid arthritis)	☐	☐
R16. Osteoporosis (thinning of the bones)	☐	☐
R17. Overweight	☐	☐
R18. Poor circulation in your legs	☐	☐
R19. Rheumatoid arthritis	☐	☐
R20. Rheumatic disease, fibromyalgia or lupus	☐	☐
R21. Stomach problem, ulcer, gastritis or reflux	☐	☐
R22. Stroke	☐	☐
R23. Thyroid disorder	☐	☐
R24. Vision problem	☐	☐
R25. Other (please fill in)		

2. Frail elderly programmes

Long-term institution admissions

Long-term institution admissions
"% of frail elderly in the programme admitted to long-term institutional care (e.g. nursing home)"

This concept can also be self-assessed, see demographics question below

What is your living situation:

- ☐ Independent, alone
- ☐ With others (partner, children, etc.)
- ☐ Care home / residential care centre since .... /........ /....... (DD/MM/YY)
- ☐ Nursing home since .... /........ /....... (DD/MM/YY)

Falls leading to hospital admissions

Falls leading to hospital admissions
"% of frail elderly in the programme in emergency room or hospital because of a fall"

ICD-10 code W00 – W19

This concept can also be self-assessed, see cost utilisation questions
