# Supplementary material for: Evaluating an integrated care pathway for frail elderly patients in Norway using multi-criteria decision analysis
Source: BMC Health Serv Res. 2021 Aug 28;21:884. doi: 10.1186/s12913-021-06805-6 (PMC8400755; doi:10.1186/s12913-021-06805-6)
Supplement: Supplementary file 2 — Additional file 2. [file 12913_2021_6805_MOESM2_ESM.docx]

**Appendix 2**

**Assumptions on unit cost estimation**

All unit costs except cost related with psychiatric hospital stay (N30) were retrieved from [44]:

N30 is collected using Samdata. The number was retrieved from [45]:

**Assumptions:**

**N20-22**
In the report the costs are originally stated per hour. It is assumed that the amount of time employees per patient is 0.3 hour. This is consistent with findings in other studies and reports.

**Cost related with Residential care and nursing homes (N28)**

In reality, costs differ dependent on whether the stay is at a nursing home or an elderly home. It is also dependent on whether it is a short term stay or a long-term stay. Unfortunately, our data does not allow us to know which of these places the patients have stayed. We have calculated the unit-cost assuming that the patients have had a short-term stay at a nursing home.

**N29**For N29 a deductible from the recipient of the service is included in the unit-cost. It is the only unit-cost of which we can safely assume that the patient has personal costs connected to the services, and the size of this cost. The deductible for other services are dependent on why the patient is receiving the services and/or the patient’s income. We do not have enough information regarding these factors.

**Medication Cost we have followed following procedures:**

1. We have collected the medication’s the Defined Daily Dose (DDD) using [42]
2. For Combination-drugs we have used 1 tablet/inhalation, which is the same amount that was used for the combination drugs listed in the document.
3. Costs for eye drops and medications for topical use (creams and gels) have not been included due to lack of DDD.
4. For some medications WHO states that the DDD is based on short term treatment. In instances where this results in large differences between the DDD and the recommended dosages in Felleskatalogen, and we have used the largest recommended dosage in Felleskatalogen. The latter amount is by far more consistent with the actual medication use, and costs, in our data.

*Table A1. Unit costs for Residential care, nursing home and home care services by municipalities (in NOK)*

| **Municipality**  **Name** | **AGA-Zone** | **Zone based on Travel time** | **N20** | **N21** | **N22** | **N28** | **N29** | **N30** | **N32** | **N33** | **N34** |
| --- | --- | --- | --- | --- | --- | --- | --- | --- | --- | --- | --- |
| Bergen | 1 | 1 | 1045 | 1045 | 1045 | 3142,30 | 3197,66 | 12826 | 521 | 521 | 764 |
| Askøy | 1 | 1 | 1045 | 1045 | 1045 | 3142,30 | 3197,66 | 12826 | 521 | 521 | 764 |
| Grimstad | 1 | 1 | 1045 | 1045 | 1045 | 3142,30 | 3197,66 | 12826 | 521 | 521 | 764 |
| Arendal | 1 | 1 | 1045 | 1045 | 1045 | 3142,30 | 3197,66 | 12826 | 521 | 521 | 764 |
| Froland | 1 | 1 | 1045 | 1045 | 1045 | 3142,30 | 3197,66 | 12826 | 521 | 521 | 764 |
| Meland | 1 | 1 | 1045 | 1045 | 1045 | 3142,30 | 3197,66 | 12826 | 521 | 521 | 764 |
| Molde | 1 | 1 | 1045 | 1045 | 1045 | 3142,30 | 3197,66 | 12826 | 521 | 521 | 764 |
| Ålesund | 1 | 1 | 1045 | 1045 | 1045 | 3142,30 | 3197,66 | 12826 | 521 | 521 | 764 |
| Birkenes | 1 | 1 | 1045 | 1045 | 1045 | 3142,30 | 3197,66 | 12826 | 521 | 521 | 764 |
| Eigersund | 1 | 1 | 1045 | 1045 | 1045 | 3142,31 | 3197,66 | 12826 | 521 | 521 | 764 |
| Tvedestrand | 1 | 1 | 1045 | 1045 | 1045 | 3142,30 | 3197,66 | 12826 | 521 | 521 | 764 |
| Lindås | 1 | 2 | 1055 | 1055 | 1055 | 3142,30 | 3197,66 | 12826 | 561 | 561 | 864 |
| Marnadal | 1 | 2 | 1055 | 1055 | 1055 | 3142,30 | 3197,66 | 12826 | 561 | 561 | 864 |
| Risør | 2 | 1 | 1025 | 1025 | 1025 | 3065,92 | 3119,44 | 12826 | 506 | 506 | 741 |
| Åmli | 2 | 1 | 1025 | 1025 | 1025 | 3065,92 | 3119,44 | 12826 | 506 | 506 | 741 |
| Surnadal | 3 | 1 | 1000 | 1000 | 1000 | 2974,25 | 3025,57 | 12826 | 488 | 488 | 717 |

*Table A2. Three-month mean costs of health and social care utilization, and informal care, in 2019 Euro*

| Cost Category | HCPC  Mean  (n=86) | Usual care (UC)  Mean  (n=41) | Difference  HCPC-UC |
| --- | --- | --- | --- |
| GPs | 128.77 | 123.87 | 4.90 |
| Nurses | 732.94 | 1070.66 | -337.73 |
| GP Assistants | 4.15 | 3.27 | 0.89 |
| Physiotherapists | 176.13 | 233.44 | -57.31 |
| Occupational therapists | 14.11 | 16.14 | -2.03 |
| Specialist Physicians | 96.66 | 69.51 | 27.14 |
| Psychologists | 20.71 | 0.00 | 20.71 |
| Hospital emergency | 12.25 | 35.03 | -22.78 |
| Hospital inpatient admissions | 426.28 | 1497.18 | -1070.90 |
| Home care services | 2025.47 | 1609.62 | 415.85 |
| Residential care/nursing homes/rehabilitation centre outpatient | 21.89 | 92.69 | -70.80 |
| Residential care/nursing homes/rehabilitation centre inpatient | 1097.97 | 3878.64 | -2780.67 |
| Medicine cost | 498.67 | 337.45 | 161.22 |
| **Subtotal costs from health care perspective** | 5421.00 | 9155.18 | **-3734.18** |
| **Informal care cost** | 809.59 | 1684.79 | **-875.20** |

Note: HCPC=integrated care, UC=usual care
