# Supplementary material for: Evaluating an integrated care pathway for frail elderly patients in Norway using multi-criteria decision analysis
Source: BMC Health Serv Res. 2021 Aug 28;21:884. doi: 10.1186/s12913-021-06805-6 (PMC8400755; doi:10.1186/s12913-021-06805-6)
Supplement: Supplementary file 3 — Additional file 3. [file 12913_2021_6805_MOESM3_ESM.docx]

**Appendix 3: Swing Weights**

**Table A3. Relative weights of the outcomes from swing weighting method (Stakeholders)**

| Outcomes | Patients  (N=158) | Partners (N=156) | Professionals (N=161) | Payers (N=122) | Policy makers  (N=180) |
| --- | --- | --- | --- | --- | --- |
| *Core-set of outcomes* |  |  |  |  |  |
| Physical functioning | 0.173 | 0.117 | 0.122 | 0.120 | 0.119 |
| Psychological well-being | 0.127 | 0.119 | 0.121 | 0.119 | 0.114 |
| Social relations & participation | 0.093 | 0.115 | 0.107 | 0.107 | 0.114 |
| Enjoyment of life | 0.133 | 0.123 | 0.124 | 0.126 | 0.121 |
| Resilience | 0.084 | 0.095 | 0.094 | 0.091 | 0.096 |
| Person-centeredness | 0.054 | 0.072 | 0.077 | 0.070 | 0.066 |
| Continuity of care | 0.057 | 0.077 | 0.076 | 0.076 | 0.079 |
| Total costs | 0.017 | 0.027 | 0.030 | 0.039 | 0.036 |
| *Programme-specific outcomes^±^* |  |  |  |  |  |
| Autonomy | 0.157 | 0.112 | 0.122 | 0.126 | 0.123 |
| Burden of medication | 0.063 | 0.079 | 0.068 | 0.070 | 0.074 |
| Informal caregiver burden | 0.042 | 0.064 | 0.059 | 0.055 | 0.057 |

Note: Numbers in parentheses (N) by stakeholders indicate the number of participants included in the online weight elicitation study for the MCDA

^±^Due to no and/or many missing responses on the programme-specific outcomes, namely, “Long-term institution admissions” and “Falls leading to hospital admissions at the baseline, and very few responded on the follow-up as well, therefore, we did not include these two outcomes in the MCDA analysis.

**Table A4. Value scores in the Multi-Criteria Decision Analysis with Swing weights (Stakeholders)**

|  | | | Patients | | Partners | | Professionals | | Payers | | Policy makers | |
| --- | --- | --- | --- | --- | --- | --- | --- | --- | --- | --- | --- | --- |
|  | Standardized performance score | | Weighted score | | Weighted score | | Weighted score | | Weighted score | | Weighted score | |
|  | HCPC | UC | HCPC | UC | HCPC | UC | HCPC | UC | HCPC | UC | HCPC | UC |
| ***Core-set of outcomes*** | | | | | | | | | | | | |
| Physical functioning | 0.708 | 0.706 | 0.122 | 0.122 | 0.083 | 0.083 | 0.086 | 0.086 | 0.085 | 0.085 | 0,084 | 0,084 |
| Psychological well-being | 0.730 | 0.683 | 0.093 | 0.087 | 0.087 | 0.082 | 0.088 | 0.082 | 0.087 | 0.081 | 0,083 | 0,078 |
| Social relationships and participation | 0.729 | 0.685 | 0.068 | 0.064 | 0.084 | 0.079 | 0.078 | 0.074 | 0.078 | 0.074 | 0,083 | 0,078 |
| Enjoyment of life | 0.732 | 0.681 | 0.098 | 0.091 | 0.090 | 0.084 | 0.091 | 0.084 | 0.093 | 0.086 | 0,089 | 0,083 |
| Resilience | 0.744 | 0.669 | 0.063 | 0.056 | 0.071 | 0.064 | 0.070 | 0.063 | 0.067 | 0.061 | 0,071 | 0,064 |
| Person-centeredness | 0.730 | 0.683 | 0.040 | 0.037 | 0.053 | 0.049 | 0.057 | 0.053 | 0.051 | 0.048 | 0,048 | 0,045 |
| Continuity of care | 0.702 | 0.712 | 0.040 | 0.040 | 0.054 | 0.055 | 0.054 | 0.054 | 0.053 | 0.054 | 0,056 | 0,057 |
| Total costs | 0.842 | 0.539 | 0.014 | 0.009 | 0.022 | 0.014 | 0.025 | 0.016 | 0.033 | 0.021 | 0,031 | 0,020 |
| **Programme-specific** ***outcomes*** | | | | | | | | | | | | |
| Autonomy | 0.664 | 0.748 | 0.104 | 0.117 | 0.074 | 0.084 | 0.081 | 0.091 | 0.084 | 0.095 | 0,081 | 0,092 |
| Burden of medication | 0.670 | 0.743 | 0.042 | 0.047 | 0.053 | 0.058 | 0.045 | 0.050 | 0.047 | 0.052 | 0,050 | 0,055 |
| Cost of informal care | 0.964 | 0.267 | 0.041 | 0.011 | 0.062 | 0.017 | 0.057 | 0.016 | 0.053 | 0.015 | 0,055 | 0,015 |
| Overall value scores  mean | | | 0.724 | 0.681 | 0.733 | 0.668 | 0.732 | 0.670 | 0.731 | 0.671 | 0.732 | 0.670 |

Note: HCPC=integrated care, UC=usual care
